# Application of a Strong Base Anion Exchange Resin for the Removal of Thiophenol from Aqueous Solution

**DOI:** 10.3390/molecules30030525

**Published:** 2025-01-24

**Authors:** Katarzyna Chruszcz-Lipska, Bogumiła Winid, Urszula Solecka

**Affiliations:** 1 Department of Oil Engineering, Faculty of Drilling, Oil and Gas, AGH University of Krakow, Mickiewicza 30 Ave., 30-059 Kraków, Poland; winid@agh.edu.pl; 2 Department of Rural Building, Faculty of Environmental Engineering and Geodesy, University of Agriculture in Krakow, Mickiewicza 24/28 Ave., 30-059 Kraków, Poland; u.solecka@urk.edu.pl

**Keywords:** thiophenol, anion exchange resin AmberLite^®^IRA402Cl, sorption, removal from water, IR spectroscopy, UV spectroscopy, DFT calculations

## Abstract

Thiophenol (synonyms: phenyl mercaptan, benzenethiol) may appear in the aquatic environment as a result of human activity. It is used as a raw material in organic synthesis in various industries for the production of dyes, pesticides, pharmaceuticals and polymers, such as polyphenylene sulfide (PPS). It may also enter water through contamination with petroleum substances (thiophenol may be present in crude oil). Due to the fact that thiophenol is toxic to living organisms, its removal from water can be a very important task. For the first time, this paper presents experimental studies of the sorption and desorption process of thiophenol on an ion exchange resin. Thiophenol sorption experiments on AmbeLite^®^IRA402 (Cl form) were tested at different pH levels (4, 7, and 9) and different ionic strengths of the aqueous solution. Its detection in water was carried out using UV spectroscopy. At pH 4, the thiophenol sorption process is basically independent of the ionic strength of the solution, but also the least effective. The sorption capacity of a thiophenol solution in distilled water is about 0.37–0.46 mg/g, for a solution with an ionic strength of 0.1 M 0.42 mg/g. At pH 7 and 9, the sorption of thiophenol from an aqueous solution is similar and definitely more effective. The sorption capacity of the thiophenol solution in distilled water is about 13.83–14.67 mg/g, and for a solution with an ionic strength of 0.1 M, it is 2.83–2.10 mg/g. The desorption efficiency of thiophenol from AmbeLite^®^IRA402 resin (washing with 4% HCl) at pH 7 is 90%, which is promising for the resin reuse process. Kinetic studies were performed and a pseudo-first-order and second-order kinetic model was fitted to the obtained experimental sorption data. In most cases, the simulation showed that the pseudo-second-order model gives a better fit, especially for the sorption of thiophenol from the solution with an ionic strength of 0.1 M. The fit of the Freundlich and Langmuir isotherm models to the experimental results indicates that the latter model provides better agreement. Analysis of the infrared spectra supported by quantum chemical calculations (DFT/PCM/B3LYP/6-31g**) confirms the experimental results observed during the sorption process. At pH 7 and 9, the thiophenol is sorbed in anionic form and—together with the ion exchange processes that occur between the dissociated thiol group and the quaternary ammonium group—an interaction between the aromatic structures of thiophenolate anions and IRA402 also takes place.

## 1. Introduction

**Figure 1:**
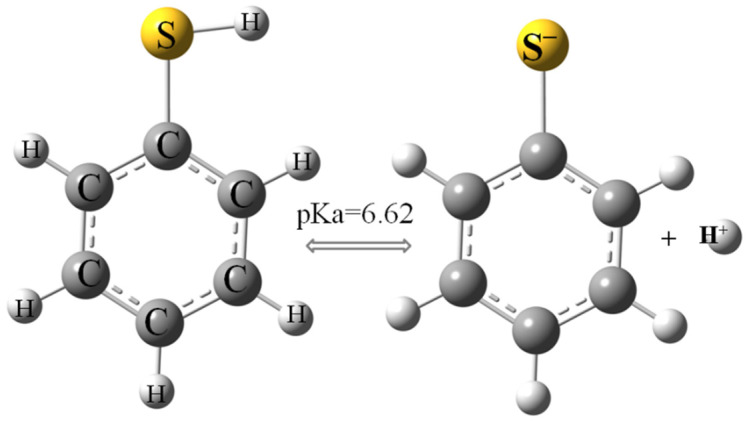
Protonated and deprotonated structure of thiophenol [4].

Thiophenol (Figure 1) is used as a raw material in organic synthesis, among other applications, for the production of pesticides, dyes, pharmaceuticals [1] and polymers, like polyphenylene sulfide (PPS), which has high mechanical, thermal and acid resistance and which is therefore widely used in various industries [2]. Thiophenol may appear in the aquatic environment, among other environments, as a result of contamination with petroleum substances because thiophenol can be found in crude oil [1,3].

Thiophenol is a chemical compound that, when released into the natural environment can contaminate water and, due to its volatility, also air. Thiophenol is toxic to organisms [5]. The average lethal dose (LD50) for fish is 0.01–0.4 mM [6]. According to the NIOSH (National Institute for Occupational Safety and Health, USA) Pocket Guide to Chemical Hazards, the permissible exposure limit for thiophenol in air is 0.5 mg/m^3^ for 15 min. The target organs for thiophenols in living organisms are the skin, eyes and respiratory system and central nervous system, liver, kidneys and spleen [7]. Many studies show methods and possibilities of detecting thiophenol in water samples [6,8,9,10,11]. However, there are only a few works that mention the detection of thiophenol in the aquatic environment. Thiophenol has been identified in municipal sewage, surface water [12] and in landfill seepage water [13].

To the authors’ knowledge, only one paper describes the removal of thiophenol from water using sepiolite [14]. Extending the literature search to the thiol group in general, it is known that mercaptans (thiols) can be removed using adsorbents such as activated carbon, zeolite molecular sieve, nanocarbon, metal–organic framework and metal oxide [15,16]. The advanced oxidation process plays an important role in the removal of mercaptans from the aquatic environment [17,18]. The globally employed technology for removing mercaptans in the oil industry is the MEROX (mercaptan oxidation) process [15,18]. However, the oxidation of mercaptans generates spent caustic, which can cause environmental problems. The treatment methods of spent caustic are: wet air oxidation, acid neutralization, advanced oxidation process, electrochemical processes and the microbiological process [18].

This study presents for the first time research on the removal of thiophenol using ion exchange resins. The application of ion exchange resins for water purification has received considerable attention over the past few decades in both the laboratory and in industry [19,20,21,22,23]. Ion-exchange resins are successfully applied for the removal of inorganic compounds such as sulfate, uranium, arsenic, bromide, bromate, boron, nitrate, perchlorate and phosphate [24,25,26,27,28,29,30,31]. There is extensive research on the use of resins in water treatment processes for the removal of natural organic matter [25,32,33,34,35,36,37,38,39]. In bench-scale batch and column experiments, strong base anion exchange resins have been tested for the removal of N-nitrosamines and N-nitrosamine precursors [40]. Certainly, the threat posed to the aquatic environment by various organic compounds, including hydrocarbons and petroleum derivatives, prompts research on the effectiveness of resins in removing these compounds [40,41,42,43]. The undeniable advantage of the ion-exchange resins is the possibility of regeneration and reuse, making them environmentally friendly. However, regeneration with a concentrated salt solution produces hazardous waste brine [44].

Although thiophenol can occur in low concentrations in waters, it is toxic to living organisms, so it is important to eliminate it from the environment. However, there are still significant knowledge gaps on how and possibly with what materials thiophenol can be removed from water. Since ion exchange resins are becoming an increasingly common material in water purification systems, this study presents for the first time research on the removal of thiophenol using an anion exchange resin (Amberlite^®^IRA402Cl). Previous research has shown that its removal from water is possible using sepiolite in the adsorption process, but only in an acidic environment [14]. In an alkaline environment, and also partially at pH 7 (pKa = 6.62), thiophenol occurs in anionic form as the thiophenolate anion [4]. Therefore, the research conducted in this study was mainly focused on the removal of thiophenolate anions, which is made possible by positively charged basic functional groups (quaternary trimethylammonium) of Amberlite^®^IRA402Cl resin.

## 2. Materials and Methods

### 2.1. Materials

**Figure 2:**
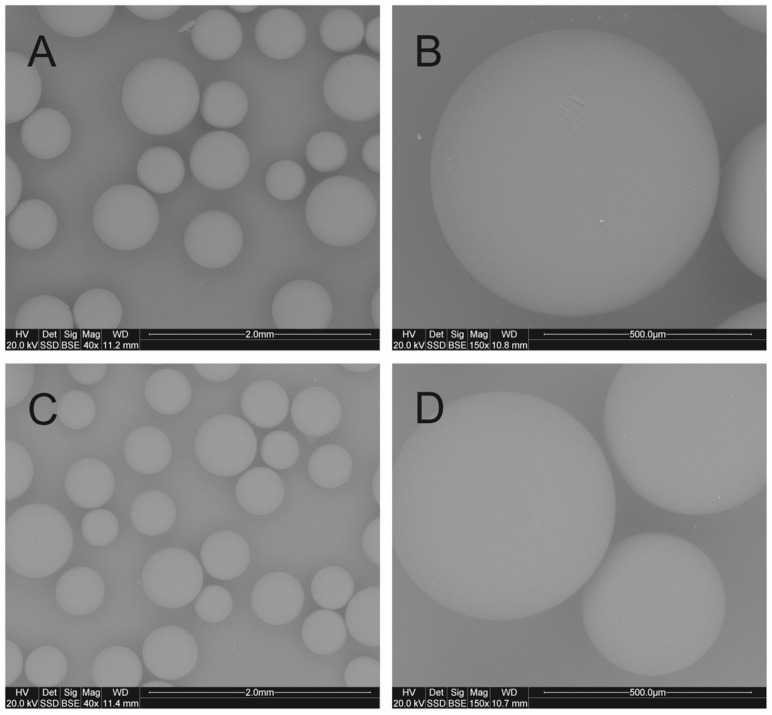
SEM images of AmberLite^®^IRA402Cl resin in distilled water (**A**,**B**) and distilled water containing 0.1 M NaCl (**C**,**D**).

Liquid thiophenol (purity 99%) was purchased from Sigma-Aldrich Chemie GmbH (Schnelldorf, Germany) and was used without further purification. The strongly basic anion exchange resin AmberLite^®^IRA402 (Cl form) was purchased from Thermo Scientific (Illkirch-Graffenstaden, France) (CAS:52439-77-7). Its characteristic properties are shown in Table 1. Additionally, resin was characterized by scanning electron microscopy (SEM) with a FEI QUANTA 200 FEG microscope. The research was carried out on samples that were originally in distilled water and distilled water containing 0.1 M NaCl. SEM images of AmberLite^®^IRA402Cl resin are shown in Figure 2. Both samples of resin were homogeneous spheres with a relatively smooth surface. No significant changes were observed at different ionic strengths.

### 2.2. IR Spectroscopy

FT-IR (Fourier Transform Infrared) spectra of all samples (the KBr pellet) were recorded using an Avatar 360 FT-IR spectrometer (Thermo Nicolet, Thermo Fisher Scientific, Waltham, MA, USA). The spectra were collected at room temperature in the spectral range of 3600–400 cm^−1^ with 256 scans at a spectral resolution of 2 cm^−1^.

### 2.3. Experimental Studies of the Adsorption of Thiophenol on AmberLite^®^IRA402l

The AmberLite^®^IRA402Cl ion exchange resin was used as a sorbent for thiophenol without any modification. Because thiophenol takes two forms in aqueous solution (protonated and deprotonated, Figure 1) the experiments were conducted at pH 4, 7 and 9. The pH was adjusted using a multifunction meter CX-601 (Elmetron, Zabrze, Poland) by adding 0.1 M NaOH or 0.1 M HCl solution. Sorption model tests were carried out using thiophenol solutions in distilled water and distilled water containing 0.1 M NaCl. The addition of sodium chloride increases the ionic strength of the solution and can reflect sorption conditions in saline water.

Detection of thiophenol in the samples during sorption experiments was performed using ultraviolet (UV) spectroscopy. UV spectra in the range of 190–400 nm were recorded using a UV-Vis spectrometer (Shimadzu UV-1700, Shimadzu Cooperation, Kyoto, Japan). The accuracy of the spectra measurement was 1 nm. The detection of thiophenol in the samples was conducted in an acidic medium by measuring absorbance at 236 nm. A detailed method of thiophenol detection using UV spectroscopy was described in a previous study [14]. In the case of experiments conducted at pH 7 and 9, the samples were acidified before measuring the UV spectrum.

Experiments examining the influence of the contact time of thiophenol with the resin during the sorption process at room temperature were carried out as follows:

(a) All experiments at pH 4, 7 and 9 were performed using 30 mg/L thiophenol solution;
(b) A total of 100 mL of appropriate thiophenol solution were added to a vessel with 1 g of AmberLite^®^IRA402Cl resin (the first screening tests showed that the sorption of thiophenol is much higher at pH 7 and 9; for thiophenol solution in distilled water, 0.2 g AmberLite^®^IRA402Cl resin were used);
(c) The samples (sorbate and sorbent) were continuously stirred on a magnetic stirrer at a speed of 100 rpm;
(d) At appropriate time intervals, mixing was temporarily stopped to take 0.5 mL samples of the solutions above the resin for UV spectroscopy tests;
(e) Sampling from above the sorbent surface was continued for approximately 140 min until equilibrium was achieved.

The experiments regarding obtaining sorption isotherms at room temperature were carried out as follows:

(a) Each time, 100 mL of thiophenol solution (with different ionic strength and different pH values) with a concentration between 1.25 and 35.00 ppm were added to the vessels with 0.5 g of AmberLite^®^IRA402Cl resin (in the case of pH 7 and 9 and thiophenol solutions (without NaCl addition), the mass of the resin equals 0.2 g);
(b) The prepared samples were shaken for 90 min on an orbital shaker at 120 rpm;
(c) After shaking, the samples were left for another 2 h;
(d) Next, the ultraviolet (UV) spectrum of the solution above the sorbent was measured.

The experiments of sorption/desorption of thiophenol at room temperature were carried out as follows:

(a) After the sorption process at pH 7 as described above (0.2 g of resin, solution of thiophenol 30 ppm) the resin was separated from the solution and washed with a small amount of distilled water;
(b) To the used resin (0.2 g), 100 mL of different eluents (distilled water, 4%, 9%, 18% hydrochloric acid, 10%NaCl, 10%NaOH) were added;
(c) The used resin, after the adding of regenerant, was shaken on an orbital shaker (120 rpm) for 90 min;
(d) The ultraviolet (UV) spectrum of the solution above the sorbent after the sorption and desorption experiments was measured.

### 2.4. DFT Calculations of Experimental IR Spectra of Thiophenol

Calculations were carried out using the Gaussian 16 software package (made available by the Cyfronet Academic Computer Centre, AGH, Kraków, Poland) [46]. Two forms of thiophenol (dissociated and undissociated form) as single molecules were studied at the DFT level using the B3LYP functional and a 6–31 g** basis set [47,48]. To best reflect the experimental conditions, i.e., an aqueous solution, the geometry optimization and IR frequencies of the molecules were calculated using the solvation model (PCM–Polarisable Continuum Model) implemented in Gaussian 16 [49,50]. For the plot of the theoretical IR spectra of the thiophenol and thiphenolate anion, Lorentzian band shapes were used with a FWHH (full width at half height) of 2 cm^−1^. The calculated harmonic frequencies were scaled down by a factor of 0.98 to account for anharmonicity effects and the limitations of the basis set [51]. The assignment of individual bands to appropriate modes in the theoretical spectra of the thiophenol and thiophenolate anion were based on direct comparison of the experimental and calculated spectra, taking into account the frequency sequence and intensity pattern visualized using GaussView 5.0 [52].

## 3. Results and Discussion

### 3.1. Kinetic Experiments

**Figure 3:**
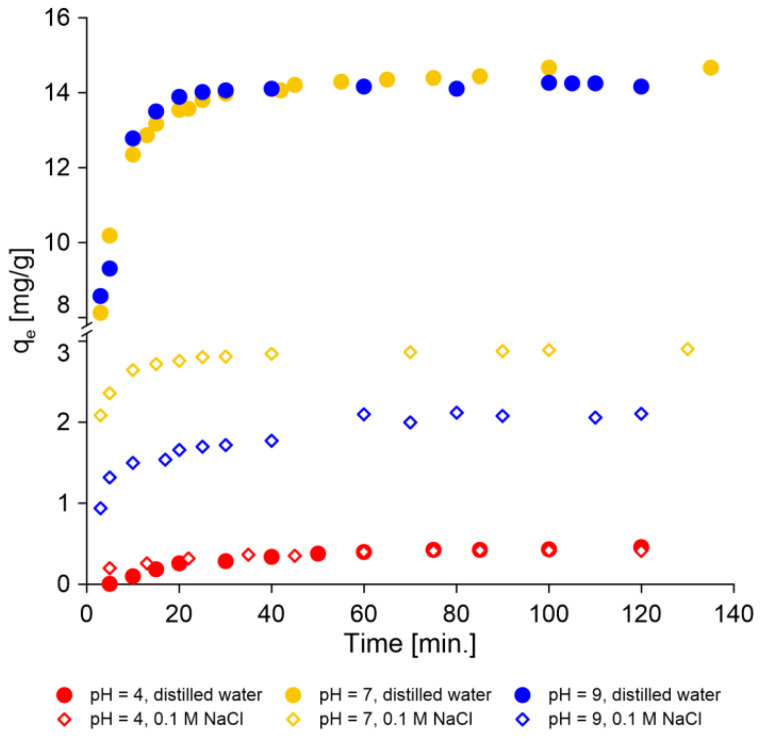
Comparison of efficiency of the sorption process of thiophenol on AmberLite^®^IRA402 (Cl form) at pH 4, 7 and 9 with different ionic strengths as a function of time (the initial concentration of thiophenol is 30 mg/L).

The evolution of thiophenol removal on AmberLite^®^IRA402 over time (kinetic experiments) was performed at different pH levels and ionic strengths of the solution. Figure 3 shows that both these physicochemical parameters strongly influenced the sorption process. The resin used in this work was a strong base anion Type I resin containing quaternary amine groups. It allows anions to be effectively removed from water, including from organic compounds. Studies using IRA 402 resin show the possibilities of the removal from the aqueous environment of even large anionic structures, such as sulfonated azo dyes: brilliant yellow [53] and Acid Blue 113 [54]. Thiophenol, depending on the pH of the aqueous solution, may also occur as an anion (Figure 1).

As can be seen in Figure 3, the sorption efficiency was actually highest when thiophenol was present mainly or completely in anionic form (pH 7 and 9) in an aqueous solution. As can be seen in Figure 3, the results of the experiment indicate that the most effective sorption was observed for thiophenol solutions prepared in distilled water. The addition of NaCl caused chloride ions to appear in the solution that definitely competed with thiophenolate ions. Therefore, the sorption of thiophenol from saline waters (ionic strength 0.1 M) was much less efficient.

The contact time significantly influences the sorption capacity of sorbent. As can be seen in Figure 3, the equilibrium state was established very quickly, after just 20 min of phase contact time at pH 7 and 9. In the case of sorption in an acidic environment (pH 4), the process was much slower and took about 80–90 min. The equilibrium state was established during the sorption of thiophenol on sepiolite for a longer period and lasted about 100 min [14]. On the other hand, the time required to reach sorption equilibrium for Acid Blue 113 on Amberlite IRA 402 was 90 min [54].

**Figure 4:**
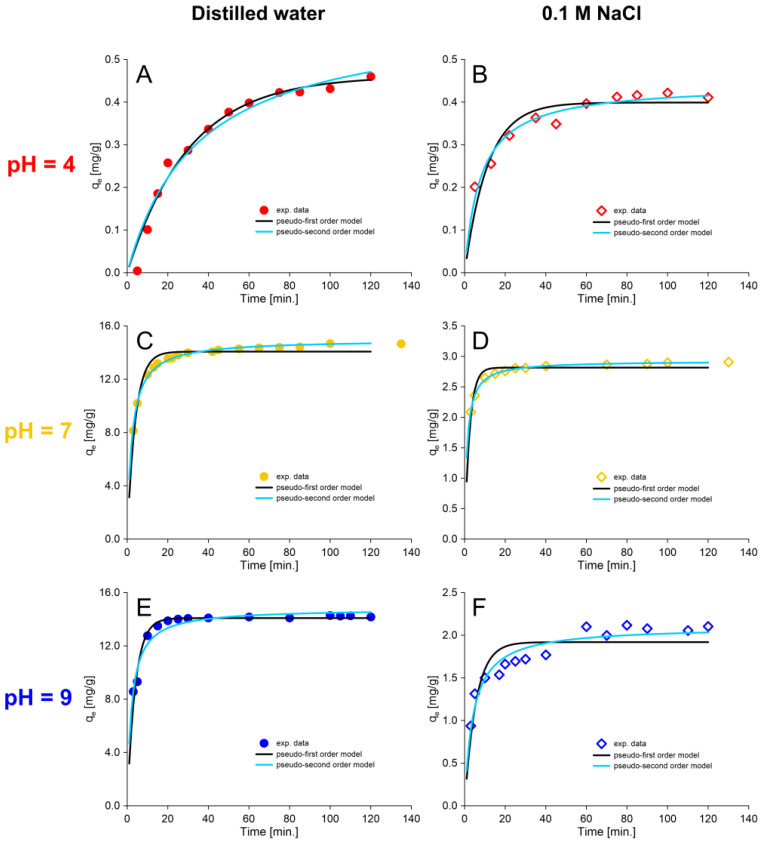
Fitting of experimental data to kinetic equations (pseudo-first-order and pseudo-second-order models): (**A**) Distilled water, pH = 4; (**B**) 0.1 M NaCl, pH = 4; (**C**) Distilled water, pH = 7; (**D**) 0.1 M NaCl, pH = 7; (**E**); Distilled water, pH = 9; (**F**) 0.1 M NaCl, pH = 9.

One possible way of simulating the sorption processes that is often used in predicting water treatment processes is sorption reaction models. Pseudo-first-order and second-order models are widely used models of sorption reactions and consist of fitting appropriate kinetic equations (Table 2) to experimental data without delving into the sorption mechanism. The parameters of the kinetic equations were determined with the nonlinear least-squares method using the Levenberg–Marquardt algorithm (the miner function in Mathcad). The results of this fitting are presented in Figure 4 and Table 2. The RMSE index (root mean squared error) and Chi^2^ (Pearson’s Chi-squared test) indicate the quality of the fit.

As can be seen in Table 2 and Figure 4, both models reflect the experimental results well. In experimental conditions that are closer to natural conditions (thiophenol solution with NaCl), the pseudo-second-order model gives a better fit. The theoretical values of sorption capacity (qe) are very close to the results of the experiments. These calculated values are 0.44, 2.93 and 2.10 mg/g for pH 4, 7 and 9, respectively (Table 2). The corresponding experimental values resulting from the trend of qe versus the sorption time (Figure 3 and Figure 4) are 0.42, 2.91 and 2.10 mg/g for pH 4, 7 and 9, respectively. The sorption process of thiophenol solution in distilled water at pH 7 also very well reflects the pseudo-second-order reaction model. The theoretical and experimental sorption capacity is 14.97 and 14.66 mg/g, respectively. The literature data indicate that in the case of sorption of organic anions onto strongly basic anion exchange resins, pseudo-second-order kinetic models usually describe experimental data better than pseudo-first-order models [55,56,57,58]. This trend includes both large anionic dye molecules [55,57] and small molecules structurally similar to thiophenol, such as phenol [56,58].

On the other hand, the kinetic trend of the sorption process of thiophenol solution in distilled water at pH 4 fits the pseudo-first-order reaction model slightly better. The theoretical sorption capacity in this condition equals 0.46 mg/g (exp. 0.44 mg/g). At pH 9 for thiophenol solution prepared from distilled water, it is problematic to determine which model actually fits the experimental data better. The RMSE index indicates that the pseudo-first-order is slightly better, but the Chi^2^ index indicates that the pseudo-second-order is slightly better (Table 2). At pH 9, the theoretical sorption capacity is 14.09 mg/g for pseudo-first-order estimation, and 14.79 mg/g for pseudo-second-order estimation (exp. 14.27 mg/g).

### 3.2. Sorption Experiments

**Figure 5:**
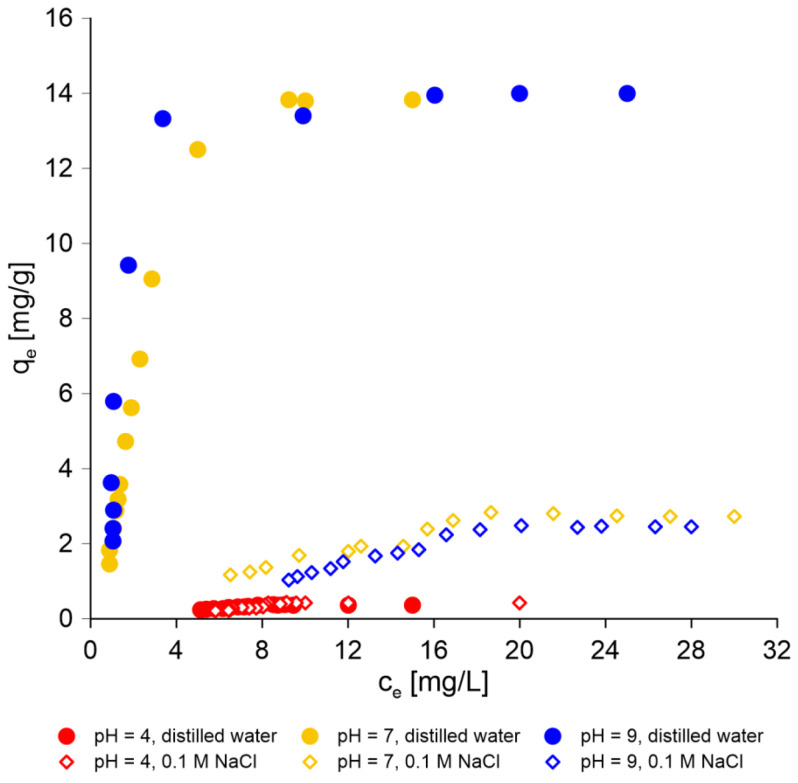
Comparison of the sorption efficiency of thiophenol on anion exchange resin IRA 402 in aqueous solutions at pH 4, 7 and 9 with different ionic strengths (distilled water, 0.1 M NaCl).

Figure 5 shows the amount of thiophenol absorbed by an IRA402, expressed as a function of the equilibrium concentration of sorbate at a room temperature. As can be seen in Figure 5, the experimental results in static conditions are consistent with those obtained in the kinetic experiment. The sorption capacity of IRA402 is highest during the sorption of thiophenol from a distilled water solution at pH 7 and 9: 13.83 and 13.95 mg/g, respectively. The sorption capacity significantly decreases to values of 2.83 mg/g (pH 7) and 2.48 mg/g (pH 9) when the ionic strength of the solution increases. Sorption in the acidic condition occurs to a very small extent. In this case, there is basically no difference due to the ionic strength of the solution. The sorption capacity of thiophenol at a concentration of 30 mg/L from the solution prepared using distilled water is 0.37 mg/g, and from the solution containing sodium chloride (ionic strength 0.1 M), it is 0.42 mg/g.

The sorption equilibrium results showing the dependence of the amount of adsorbed substance on the concentration of the adsorbate at a constant temperature (Figure 5) were fitted to the Langmuir and Freundlich isotherm models [59,60]. The study of sorption isotherms is important from the point of view of the design and operation of sorption systems [61]. The parameters of the isotherm equations were determined in the same way as in the kinetic experiments by using the nonlinear least-squares method and the Levenberg–Marquardt algorithm. The results of this fitting are presented in Figure 5 and Table 3.

**Figure 6:**
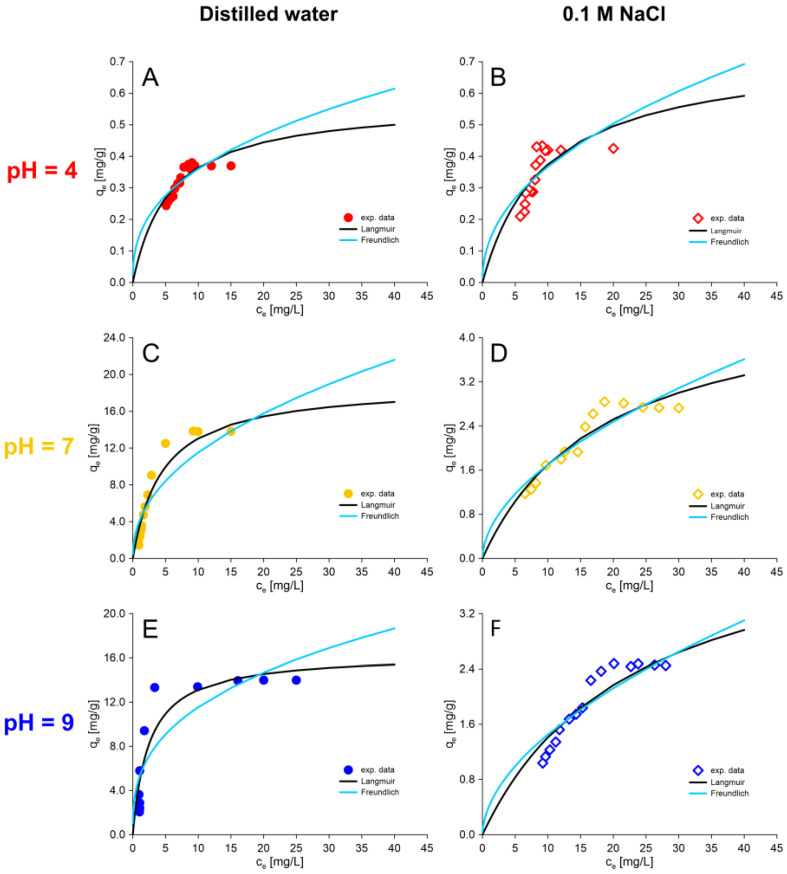
Isotherm models for experimental data of the thiophenol sorption process on AmberLite^®^IRA402Cl at pH 4, 7 and 9 with different ionic strengths: (**A**) Distilled water, pH = 4; (**B**) 0.1 M NaCl, pH = 4; (**C**) Distilled water, pH = 7; (**D**) 0.1 M NaCl, pH = 7; (**E**); Distilled water, pH = 9; (**F**) 0.1 M NaCl, pH = 9.

The sorption equilibrium results that show the dependence of the amount of adsorbed substance on the concentration of the adsorbate at a constant temperature (Figure 4) were fitted to the Langmuir and Freundlich two isotherm models [59,60]. The study of sorption isotherms is important from the point of view of design and operation of sorption systems [61]. The parameters of the isotherm equations were determined in the same way as in the kinetic experiments using the nonlinear least-squares method and the Levenberg–Marquardt algorithm. The results of this fitting are presented in Figure 6 and Table 3.

Analysis of the estimated values of the fitting indexes of the theoretical equations (RMSE, Chi^2^) (Table 3) shows that the process of thiophenol sorption from the solution is better described by the Langmuir isotherm in each case. According to the literature, similar results have been reported for the sorption of other organic sorbates on strong base anion exchange resin. The Langmuir isotherm better fits the experimental results during the sorption of Acid Blue 113 dye on Amberlite IRA 402 [54], Acid Orange 7, Acid Orange 10 and Acid Red 88 dyes on Amberlite IRA-458 [62] and phenol on Amberlyst A26 [63].

The estimated values of the QL parameters (maximum sorption capacity) from the Langmuir equations are in good agreement with the results of the experiment and in all cases slightly higher than the experimental values. For the thiophenol solutions prepared using distilled water, they are 0.571 mg/g for pH 4 (exp. 0.37 mg/g), 18.936 mg/g for pH 7 (exp. 13.83 mg/g) and 16.351 mg/g for pH 9 (exp. 13.95 mg/g). For the thiophenol solutions with 0.1 M ionic strength (with addition of NaCl), they are 0.734 mg/g for pH 4 (exp. 0.42 mg/g), 4.853 mg/g for pH 7 (exp. 2.83 mg/g) and 4.684 mg/g for pH 9 (exp. 2.48 mg/g).

### 3.3. Desorption Process of Thiophenol from AmberLite^®^IRA402 Resin

One of the key points of the planned industrial use of ion exchange resin is its effective regeneration and readiness for reuse, so that the installation can be economically viable and environmentally friendly [64]. The recycling of used resins is a problem studied primarily on a laboratory scale. During experiments, the appropriate washing solution was selected so that the effects of the desorption process was as high as possible. The suppliers of Amberlite^®^IRA402Cl resin present different regenerants for their products in the product data sheet, for example Lenntech [65] and BSBL [66] propose NaOH solution (2–4%), and Dupont [67] proposes regeneration with solutions, where the main component is 10% NaCl (in the composition also HCl, NaOH and H_2_SO_4_ are listed). In turn, Marin and Stanculescu carried out desorption of acid blue 113 previously adsorbed on Amberlite^®^IRA402 using hydrochloric acid (molar concentration of HCl 1.0–7.0 M) [54].

**Figure 7:**
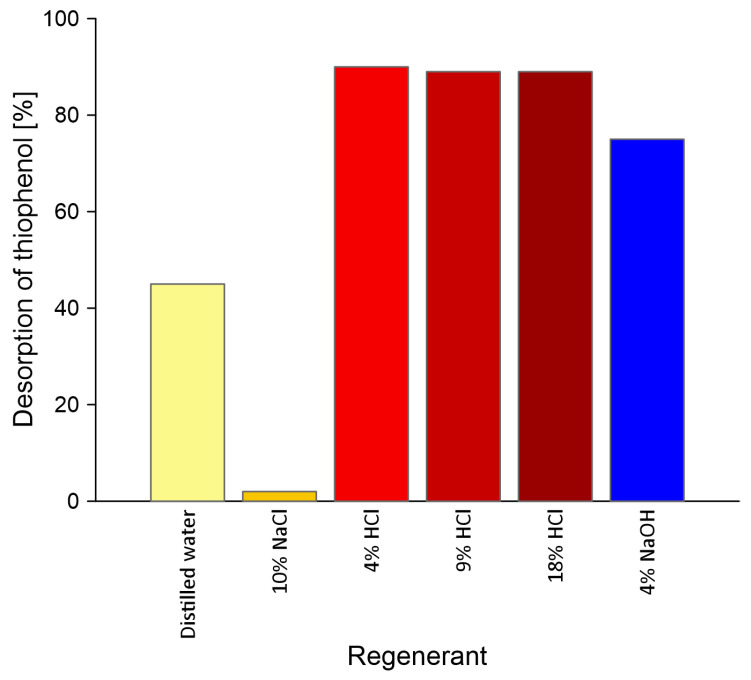
Influence of regenerant type on the desorption of thiophenol from Amberlite^®^IRA402 resin at pH 7.

Due to the discrepancies in the used regeneration solutions, in this study, we conducted a sorption experiment of thiophenol at pH 7. Sorption was the most effective in that condition. The sorption process was performed using 0.2 g of resin and thiophenol solution with a concentration of 30 ppm. Then, the process of desorption of thiophenol under the influence of various reagents was performed. The reagents used were distilled water, HCl (4%, 9%, 18%), 10% NaCl and 4% NaOH. Figure 7 shows the influence of the regenerant on the desorption of thiophenol from the Amberlite^®^IRA402 resin. The thiophenol desorption expressed the ratio of the mass of thiophenol in the solution after the desorption experiment (with different regenerants) to that mass of thiophenol which was sorbed during sorption (in a percentage). As can be seen in Figure 7, the type of regenerant was very important in this case to obtain the best regeneration results. In our experiment, when applying the desorption test, the amount of thiophenol in solution above the resin was the highest (ca. 90%) in the case of hydrochloric acid. This means that at pH 7 the ion exchange process was mainly responsible for the removal of the thiophenol from the solution. During 90 min of shaking with 10% NaCl solution, practically no desorption of thiophenol from the resin was observed. This means that thiophenol was strongly bound to it. Paulino and Afonso also observed that strong basic anion-exchange resins strongly retained sulphur compounds (with S^2−^ ions) [68]. It can be also seen that the desorption of thiophenol did not change with increasing HCl concentrations from 4 to 18%, which indicates that at pH 7 ion exchange is not the only mechanism for removing thiophenol from an aqueous solution and a small part of it is adsorbed on the resin surface.

### 3.4. Investigation of the Sorption Process of Thiophenol on AmberLite^®^IRA402 Using FT-IR Spectroscopy

Various vibrational spectroscopy techniques, such as FT-IR spectroscopy, are often used to obtain valuable information concerning the sorption process. The analysis of changes in the infrared spectrum of sorbent during this process is very frequently helpful in understanding it. IR studies enable the identification of functional groups which are characteristic of pure sorbate and sorbent and which indicate the participation of these specific functional groups in sorption interaction after sorption. The use of infrared spectroscopy has been described in the literature, to both characterize IRA 402 resin and to confirm that the surface of this resin has been modified by various chemical syntheses [63,69,70,71]. Infrared spectroscopy has also been used to characterize possible interactions between the resin and the adsorbed substance [54,71]. The analysis of experimental and theoretical vibrational spectra (IR and Raman) of the sorbate (thiophenol) has been described many times. Authors have most often described the spectrum of thiophenol in undissociated form and compared it with the spectra of the obtained complexes with noble metals (Ag and Au) [72,73,74,75,76]. This ability to bind to metals makes thiols useful as selective sorbents for removing heavy metals from the aqueous environment [77,78].

**Figure 8:**
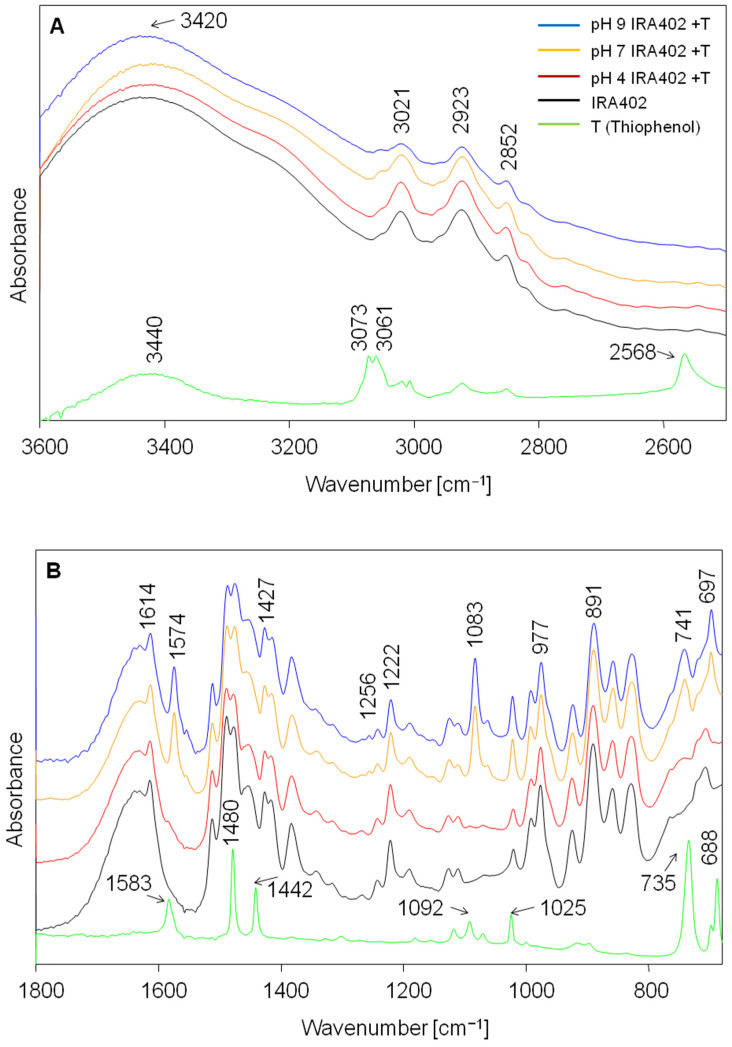
Experimental FT-IR spectra of thiophenol, pure ion exchange resin IRA 402, and IRA 402 after sorption of thiophenol (at pH 4, 7 and 9) in the spectral ranges of 3600–2550 (**A**) and 1800–680 cm^−1^ (**B**).

Figure 8 shows a comparison of the FT-IR spectra of thiophenol, pure ion exchange resin IRA 402 and IRA 402 after the sorption of thiophenol (at pH 4, 7 and 9). Infrared spectroscopy studies confirm that IRA 402 resin adsorbs very small amounts of thiophenol in acidic media. This is evidenced by very small changes in the spectrum of the resin, which was in contact with thiophenol for 2 h at pH 4. The most intense bands originating from thiophenol at 1583, 1480, 1442, 735 and 688 cm^−1^ slightly influenced the shape of the bands in the infrared spectrum of the resin after contact with thiophenol.

Changes are evident in the infrared spectrum of IRA 402 resin after the sorption process at pH 7 and 9 (Figure 8). In addition, they clearly indicate that thiophenol was adsorbed in anionic form in these conditions. The presence of the thiophenolate ion is manifested by a shift of the band at 1583 cm^−1^ to 1574 cm^−1^. This IR signal is associated with the C-C stretching vibrations in the benzene ring and appears in the dissociated form of thiophenol at a frequency of ca. 1575 cm^−1^ [76,79,80]. Figure 8 shows that the band at 1092 cm^−1^ (thiophenol) shifts to lower wavenumbers to 1083 cm^−1^ (thiophenolate adsorbed on IRA 402 at pH 7 and 9). This band mainly corresponds to C-S stretching vibration [73,76]. Significant changes in the spectra are also observed around 700 cm^−1^. The intense bands at 735, 697 and 689 cm^−1^ seen in the spectrum of undissociated thiophenol are not observed after the thiophenol sorption process on the ion exchange resin (pH 7 and 9, Figure 8). These bands come from C-H out-of-plane bending vibrations (735 cm^−1^), ring in-plane deformations (699 cm^−1^) and ring out-of-plane deformations (689 cm^−1^) [77]. However, in the experimental spectrum of resin IRA402 with adsorbed thiophenol (pH 7 and 9) strong bands at 741 and 697 cm^−1^ are observed. Additionally, a weak band appears at 1256 cm^−1^, which is most likely due to changes upon sorption in the vibrations associated with the amine functional group of the resin. In the pure resin Amberlite@IRA402, the bands in this spectral range (at 1268 and 1243 cm^−1^) are connected mainly to the stretching vibration of the C-N bond and the C-H bending vibration in –CH_2−_ group between the ring and the N(CH_3_)_3_ group [81]. The second explanation for the appearance of the band at 1256 cm^−1^ could be the change in the C-H vibrations in the aliphatic chain of the resin [80].

**Figure 9:**
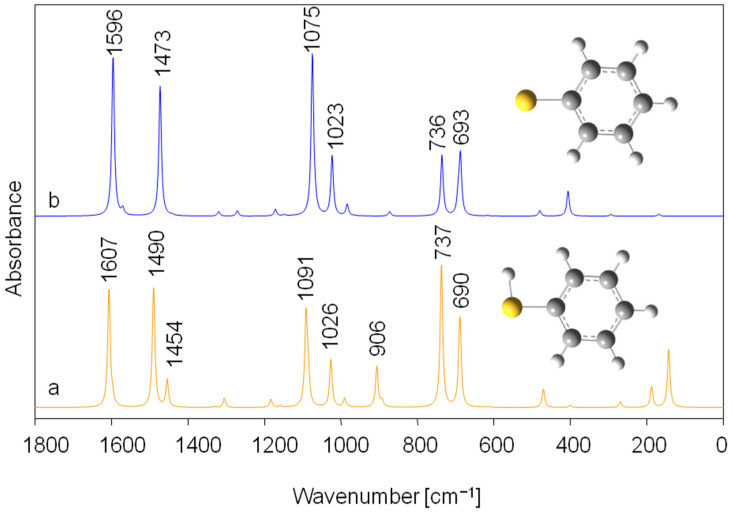
Theoretical (DFT/PCM/B3LYP/6-31g**) infrared spectra of thiophenol (a) and thiophenolate anion (b) in the spectral range of 1800–0 cm^−1^.

The obtained theoretical (DFT/PCM/B3LYP/6-31g**) infrared spectra for dissociated and undissociated thiophenol structure (Figure 9) are consistent with the results of the experiment. It should be noted that, to the best of our knowledge, calculations of the infrared spectra of thiophenol and thiophenolate ion using a solvation model (PCM) have not yet been reported in the literature. The strong signal associated with the C-C stretching vibrations in the benzene ring seen in the theoretical spectrum of thiophenol at 1607 cm^−1^ also shifts towards lower frequencies and appears in the calculated spectrum of the dissociated form of thiophenol at 1596 cm^−1^ (experimental counterparts are at 1583 and 1574 cm^−1^, respectively, Figure 9). The mixed vibration originating from C-H in-plane bending (in the ring) and C-C stretching is seen in the theoretical spectrum of the thiophenol and thiophenolate anion at 1490 and 1473 cm^−1^, respectively. The strong intensity band that mainly originates from C-S stretching vibration also shifts towards lower wavenumbers. This is observed for thiophenol at 1091 cm^−1^ and for the thiophenolate anion at 1075 cm^−1^ (experimental counterparts are at 1092 and 1083 cm^−1^, respectively, Figure 9).

The strong bands in the experimental spectrum of thiophenol in the range of 750–680 cm^−1^ (at 735 and 688 cm^−1^ (699 cm^−1^ shoulder)) are present in the theoretical spectrum at 737 and 690 cm^−1^ (689 cm^−1^ shoulder). In the case of the anionic form of thiophenol, these bands lose intensity but only slightly change their positions (Figure 9). Therefore, in the spectrum of the thiophenolate anion adsorbed on the resin at pH 7 and 9 (Figure 8), there should be no change in the frequency of the bands in this spectral region relative to the spectrum of pure undissociated thiophenol. However, the changes are clearly visible and cannot be explained solely by the presence of thiophenol as the thiophenolate ion. As mentioned earlier, strong bands at 741 and 697 cm^−1^ are observed in these spectra (pH 7 and 9, Figure 8). This indicates that in-plane ring deformation, out-of-plane ring deformation and out-of-plane C-H bending vibrations of thiophenol do not vibrate completely freely after the sorption process. This finding indicates that—together with the ion exchange process that occurs between the dissociated thiol group and the quaternary ammonium group—the interaction (van der Waals and/or π-π) between the aromatic structures of thiophenolate anion and IRA402 also takes place.

## 4. Conclusions

The conducted research demonstrates for the first time the possibility of the successful removal of thiophenol—a substance that is toxic to living organisms—by sorption from an aqueous solution on an ion exchange resin. The sorption process of thiophenol on Amberlite^®^IRA402Cl resin differs significantly according to the pH and the ionic strength of the water solution. Since Amberlite^®^IRA402Cl is a strong basic anion exchange resin whose positively charged basic functional groups (quaternary trimethylammonium) give the resin an affinity for anions, the sorption of thiophenol is much more efficient when it is present in water solution in the anionic form. The sorption capacity of the thiophenol solution (in distilled water) is in the range of 13.83–14.67 mg/g at pH 7 and 13.95–14.27 mg/g at pH 9. In an aqueous solution with an ionic strength of 0.1 M, the sorption efficiency decreases. The sorption capacity of the thiophenol solution (with an ionic strength of 0.1 M) is in the range of 2.83–2.91 mg/g at pH 7 and 2.10–2.48 mg/g at pH 9. At pH 4, the thiophenol sorption process is much less effective, but it is basically independent of the ionic strength of the solution. The sorption capacity is about 0.37–0.46 mg/g for a solution of thiophenol in distilled water and is 42 mg/g for a solution with an ionic strength of 0.1 M. The desorption efficiency of thiophenol from AmbeLite^®^IRA402 resin after one cycle sorption/desorption is 90% using 4% HCl as regenerant, which is important in the context of the reuse process of this resin.

Theoretical models of kinetic equations of sorption reactions (pseudo-first-order and pseudo-second-order kinetic models) and isotherm models were fitted to experimental data. In most cases, the pseudo-second-order model gave a better fit, especially for the sorption of thiophenol from the solution with an ionic strength of 0.1 M. The fit of the theoretical isotherm models to the experimental results in all investigated conditions indicated that the Langmuir isotherm provides better agreement than the Freundlich isotherm. The estimated values of maximum sorption capacity from the Langmuir equations are in good agreement with the result of the experiment and in all cases slightly higher than the experimental values. For thiophenol solutions prepared using distilled water, they are 0.571 mg/g, 18.936 mg/g and 16.351 mg/g for pH 4, 7 and 9, respectively. For thiophenol solutions with 0.1 M ionic strength, they are 0.734 mg/g, 4.853 mg/g and 4.684 mg/g for pH 4, 7 and 9, respectively.

The sorption process was also studied using infrared spectroscopy. To the best of our knowledge, simulation (DFT/PCM/B3LYP/6-31g**) of the IR spectra of the dissociated and undissociated forms of thiophenol in aqueous solution were performed for the first time. Quantum chemical calculations were performed to better understand the interactions between the sorbate and the sorbent. The analysis of the IR spectra of the Amberlite^®^IRA402Cl resin measured before and after sorption process confirmed that at pH 7 and 9—besides the ion exchange process that takes place between the dissociated thiol group and the quaternary ammonium group of resin—there is also an interaction between the aromatic structures of the thiophenolate anion and IRA402.

## Figures and Tables

**Table 1:** Characteristic properties of AmberLite*^®^*IRA402C [45].

Amberlite^®^IRA402Cl	
Type	Strong base anion Type I
Copolymer	Styrene-divinylbenzene
Functional Group	Trimethylammonium
Ionic Form as Shipped	Cl^−^
Total Exchange Capacity	≥1.20 eq/L (Cl^−^ form)
Water Retention Capacity	49.0–59.0% (Cl^−^ form)
Particle Diameter	600–750 μm
Particle Density	1.07 g/mL
Shipping Weight	670 g/L

**Table 2:** Kinetic parameters for sorption process of thiophenol on Amberlite^®^IRA402.

pH	Solvent	Pseudo-First Order Model q_t_ = q_e_(1 − e^k^_1_^t^) Plot q_e_ Versus t				Pseudo-Second Order Model $q_{t}=\frac{k_{2}\cdot {q_{e}}^{2}\cdot t}{1+k_{2}\cdot q_{e}\cdot t}$ Plot q_e_ Versus t			
		q_e_	k_1_	Chi^2^	RMSE	q_e_	k_2_	Chi^2^	RMSE
4	distilled water	0.460	0.033	0.074	0.024	0.610	0.048	0.087	0.027
	0.1 NaCl	0.400	0.088	0.035	0.027	0.440	0.291	0.009	0.016
7	distilled water	14.070	0.252	0.255	0.429	14.970	0.029	0.028	0.136
	0.1 NaCl	2.820	0.408	0.025	0.074	2.930	0.287	0.001	0.012
9	distilled water	14.090	0.255	0.229	0.380	14.790	0.031	0.222	0.435
	0.1 NaCl	1.920	0.180	0.260	0.178	2.100	0.116	0.090	0.103

q_e_—equilibrium sorption capacity; k_1_—pseudo-first-order rate constant; k_2_—pseudo-second-order rate constant; Chi^2^—Pearson’s Chi-squared test; RMSE—root mean squared error.

**Table 3:** The results of fitting the Langmuir and Freundlich isotherm models to experimental data for thiophenol sorption on AmberLite^®^IRA402.

Isotherm Model	pH	Solvent	Constants		Quality of Fitting	
			K_L_/K_F_	Q_L_/1/n	Chi^2^	RMSE
Langmuir $q_{e}=\frac{Q_{L}\cdot K_{L}\cdot c_{e}}{1+K_{L}\cdot c_{e}}$ plot $q_{e}$ versus $c_{e}$	4	distilled water	0.175	0.571	0.025	0.023
		0.1 M NaCl	0.104	0.734	0.121	0.051
	7	distilled water	0.221	18.936	4.567	1.337
		0.1 M NaCl	0.054	4.853	0.223	0.192
	9	distilled water	0.404	16.351	6.677	1.966
		0.1 M NaCl	0.043	4.684	0.416	0.226
Freundlich $q_{e}=K_{F}\cdot c_{e}^{1/n}$ plot $q_{e}$ versus $c_{e}$	4	distilled water	0.148	0.386	0.033	0.026
		0.1 M NaCl	0.128	0.458	0.144	0.055
	7	distilled water	4.05	0.454	10.546	2.193
		0.1 M NaCl	0.488	0.542	0.319	0.227
	9	distilled water	5.198	0.346	10.845	2.578
		0.1 M NaCl	0.411	0.548	0.571	0.267

q_e_—equilibrium sorption capacity; K_L_—Langmuir constant; c_e_—equilibrium concentration of sorbate in solution; Q_L_—maximum sorption capacity; K_F_—Freundlich constant; 1/n is the heterogeneity factor; Chi^2^—Pearson’s Chi-squared test; RMSE—root mean squared error.

## Data Availability

Data available on request from corresponding author.
